# Thermal Metamaterials with Configurable Mechanical Properties

**DOI:** 10.1002/advs.202406116

**Published:** 2024-09-03

**Authors:** Yihui Wang, Wei Sha, Mi Xiao, Liang Gao

**Affiliations:** ^1^ State Key Laboratory of Intelligent Manufacturing Equipment and Technology Huazhong University of Science and Technology Wuhan 430074 China

**Keywords:** configurable mechanical properties, data‐driven, thermal metamaterials

## Abstract

Thermal metamaterials are typically achieved by mixing different natural materials to realize effective thermal conductivities (ETCs) that conventional materials do not possess. However, the necessity for multifunctional design of metamaterials, encompassing both thermal and mechanical functionalities, is somewhat overlooked, resulting in the fixation of mechanical properties in thermal metamaterials designed within current research endeavors. Thus far, conventional methods have faced challenges in designing thermal metamaterials with configurable mechanical properties because of intricate inherent relationships among the structural configuration, thermal and mechanical properties in metamaterials. Here, a data‐driven approach is proposed to design a thermal metamaterial capable of seamlessly achieving thermal functionalities and harnessing the advantages of microstructural diversity to configure its mechanical properties. The designed metamaterial possesses thermal cloaking functionality while exhibiting exceptional mechanical properties, such as load‐bearing capacity, shearing strength, and tensile resistance, thereby affording mechanical protection for the thermal metadevice. The proposed approach can generate numerous distinct inverse design candidate topological functional cells (TFCs), designing thermal metamaterials with dramatic improvements in mechanical properties compared to traditional ones, which sets up a novel paradigm for discovering thermal metamaterials with extraordinary mechanical structures. Furthermore, this approach also paves the way for investigating thermal metamaterials with additional physical properties.

## Graphical abstract

Utilizing AI‐driven data augmentation and deep generative models, a novel approach is pioneered to design thermal metamaterials with customizable mechanical properties. By leveraging latent variables and clustering techniques, diverse topological functional cells are achieved exhibiting superior thermal cloaking and mechanical strength. The methodology promises to advance metamaterial design, offering new avenues for enhancing metamaterial functionality.

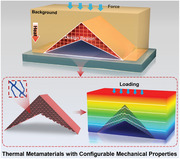

## 1 Introduction

Metamaterials, as artificially designed composite materials, not only exhibit remarkable properties absent in natural materials but can also be tailored according to design requirements, which have revolutionary development prospects in thermotics,^[^ ^1^, ^2^ ^]^ electromagnetics,^[^ ^3^, ^4^ ^]^ optics,^[^ ^5^, ^6^, ^7^, ^8^, ^9^, ^10^, ^11^ ^]^ acoustics,^[^ ^12^, ^13^ ^]^ and mechanics.^[^ ^14^, ^15^ ^]^ As a member of the metamaterial family, thermal metamaterials have powerful regulation capabilities of heat flow compared to natural materials, and they can be designed with special structures to achieve thermal management, which has received extensive research and attention.^[^ ^16^, ^17^, ^18^, ^19^, ^20^, ^21^, ^22^, ^23^, ^24^, ^25^ ^]^ Mixing different natural materials with divergent thermal conductivities stands as a foremost approach to surpass the effective thermal conductivities (ETCs) of naturally occurring materials. By orchestrating these materials with precision at the micro or nano‐scale, it becomes feasible to engineer structures endowed with tailored thermal properties, thereby enabling an unparalleled mastery over the heat flow.^[^ ^26^, ^27^, ^28^ ^]^ However, in engineering practice, researchers have primarily focused on addressing thermal‐related issues, while the multifunctional requirements and multiphysics design that simultaneously satisfy both thermal and mechanical functionalities have not received sufficient attention. For instance, how to maintain the thermal functionality of metamaterials while achieving additional extraordinary mechanical functionalities or configurable mechanical properties (such as mechanical cloaking, negative Poisson's ratio, energy absorption, and high load‐bearing capacity), which is a pressing issue in practical applications but often overlooked in current researches. The existing study^[^ ^29^ ^]^ has only addressed displacement field manipulation problem under thermal loads, neglecting considerations for multifunctional design of thermal metamaterials, which is evidently insufficient.

To tackle this issue, we have discovered that the utilization of diversified topological functional cells (TFCs) enables thermal metamaterials to achieve configurable mechanical performance. Essentially, the mechanical properties of thermal metamaterials are naturally determined by the designed structure that satisfies the desired ETC. It is found that for a given ETC, the inverse‐designed structures can exhibit variability,^[^ ^30^ ^]^ thus the mechanical properties of these thermal metamaterials can potentially be configurable by selecting different TFCs according to specific requirements. In research, conventional methodologies for designing thermal metamaterials typically rely on numerical optimization to craft microstructures meeting the desired ETC. However, these methods often fail to fully exploit and consider the potential advantage offered by microstructural diversity, since addressing this issue typically involves considering the coupling of multiple physical fields and multi‐objective optimization. The complex relationship between structural configuration and multifunctional performance makes it challenging for traditional methods to employ optimization algorithms effectively.^[^ ^31^ ^]^ Additionally, with the increase in design freedom and complexity, formidable computational costs pose another significant challenge.

With the advancement of artificial intelligence (AI), data‐driven techniques offer promising innovation in metamaterial design. Leveraging deep learning (DL) models, researchers are able to provide novel solutions for metamaterials with minimal computational costs and high flexibility. Recent works have applied DL with endeavors extending to various scenarios, such as thermal metamaterials,^[^ ^32^, ^33^, ^34^, ^35^ ^]^ mechanical metamaterials,^[^ ^36^, ^37^, ^38^ ^]^ plasmonic metamaterials, and^[^ ^39^ ^]^ nanophotonic particles.^[^ ^40^, ^41^ ^]^ Nevertheless, some of these studies simply treat metamaterial design as a one‐to‐one mapping regression problem between properties and structures, thereby underutilizing the capability of DL models to establish one‐to‐many mappings. Despite the acknowledgment of microstructural diversity in some investigations,^[^ ^42^, ^43^ ^]^ there exists a gap in exploring how to effectively capitalize on this diversity to address multifunctional design requisites in engineering applications. Additionally, from a data‐driven perspective, while extensive efforts are made to collect increasingly larger volumes of data, current researches have attracted scant attention to the richness of information within the data,^[^ ^44^ ^]^ let alone how to fully leverage the advantages of data diversity and their applications.

**Figure 1:**
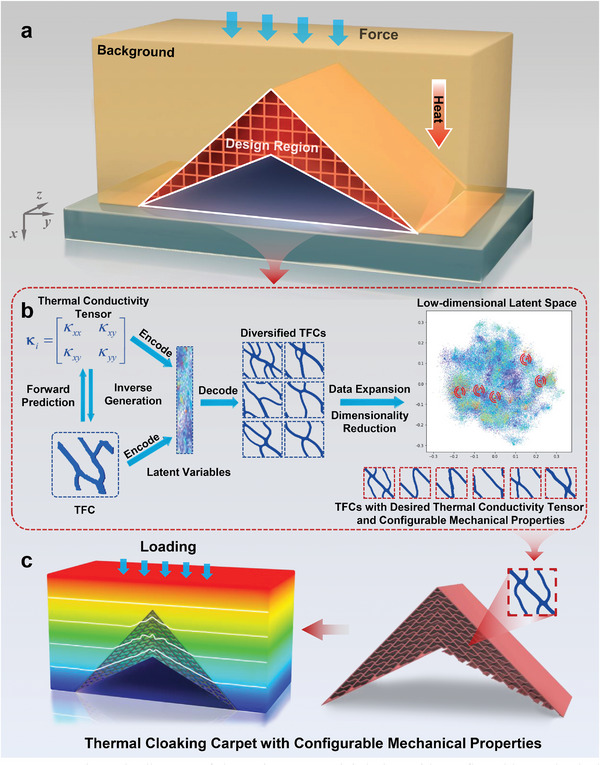
Schematic diagram of thermal metamaterial design with configurable mechanical properties. a) Displays the application scenario of thermal metamaterials. The red grid region represents the design domain, with the dark blue area below representing the protected object. The thermal metamaterial is embedded within a thermally conductive background material, depicted by the red arrows indicating the downward heat flow. Additionally, the entire thermal metadevice is subjected to vertical pressure force. b) Proposed data‐driven design of diversified TFCs schematic. TFCs and their corresponding thermal properties are encoded as latent variables, which can be decoded into diversified TFCs through sampling from the latent variables. The forward path is designed as a deterministic prediction process, encompassing the prediction of thermal and mechanical properties. Through a series of data augmentation operations and retraining processes, the scatter plot represents a schematic diagram of the latent space after data dimensionality reduction. Each point represents a sample, with their colors indicating different mechanical properties. By sampling at specific locations in the latent space, various TFCs with same thermal properties but different mechanical properties (such as extremal *C*_11_, *C*_22_, *C*_33_, and elastic properties) can be obtained. c) Represent the unique features of the designed thermal carpet with superior load‐bearing capacity and perfect thermal cloaking functionality.

To address all these challenges, in this work, we achieve the thermal metamaterials with configurable mechanical properties (**Figure** **1**a) by proposing a modified deep generative model based on conditional variational autoencoder (CVAE). This model can solve the one‐to‐many mapping problem by introducing latent variables as probabilistic representation (Figure 1b), and greatly enriches the diversity of TFCs through data expansion technique. Subsequent iterations of model retraining by using the self‐supervised learning strategy, performing data dimensionality reduction, and clustering operations, we can pinpoint polytype metamaterial microstructures within the latent space possessing similar thermal properties yet exhibiting varied mechanical performances, thereby naturally designing thermal cloaking carpet with configurable mechanical properties as depicted in Figure 1c. The designed metamaterial possesses thermal cloaking functionality while exhibiting exceptional structural resistance that is capable of powerful load‐bearing capacity. In accordance with the characteristics of configurable mechanical properties, similar thermal cloaking metamaterials can also be designed to have mechanical properties of shear and tensile resistance, thereby affording thermal and mechanical protection for the metadevice in applications, paving the way for the discovery of thermal metamaterials with extraordinary mechanical structures.

## 2 Results and Discussion

### 2.1 Design of Thermal Cloaking Carpet with Configurable Mechanical Properties

The concept of thermal cloaking has emerged as an area of considerable interest, driven by its promising applications in thermal management and energy conservation. Nevertheless, conventional thermal cloaking metamaterials are encumbered by inherent limitations in mechanical robustness, thereby constraining their versatility in real‐world scenarios. Here, we study endeavors to explore the intricate design of a thermal cloaking carpet, which transcends conventional approaches by not only achieving thermal cloaking but also imparting configurable mechanical properties tailored to diverse situations.

#### 2.1.1 Design of Thermal Cloaking Carpet Based on Regionalized Scattering Cancellation Method

Drawing inspiration from the scattering cancellation method,^[^ ^45^, ^46^, ^47^ ^]^ the regionalized scattering cancellation method^[^ ^48^ ^]^ has been developed and successfully applied to the design of thermal metamaterials. This method subdivides the thermal metamaterial into multiple subregions and computes the solutions to the heat conduction equation under specific boundary constraints, thereby establishing the relationships between the required ETCs of each subregion. Specifically, according to Fourier's law equation **q** = −**κ** ·∇**T**, one can derive the relationship between the heat flux vectors across the interface (i.e., from Region *i* to Region *i*+1) of two materials with different thermal conductivity tensors $\kappa =[\begin{array}{cc} \kappa_{xx} & \kappa_{xy}\\ \kappa_{yx} & \kappa_{yy} \end{array}]$ as follows:

(1) $$q^{\left(in,out\right)}=-\kappa^{\left(i,i+1\right)}\cdot \nabla T^{\left(i,i+1\right)}$$

where **κ** *^i^* and **T** *^i^* represent the thermal conductivity tensor and temperature distribution of Region *i*.

According to Equation (1), it becomes apparent that, within a defined temperature gradient, deliberate engineering of the thermal conductivity tensors **κ** for two distinct materials enables precise control over the direction of heat flux. Leveraging this approach, we can devise thermal cloaking metamaterials within the design domain. As illustrated in Figure S1 (Supporting Information), we partition the design area into several regions through multiple interfaces. The macroscopic configuration of the thermal cloaking metamaterial is determined by four geometric parameters, |*AO*|, |*AD*|, θ_1_, and θ_2_. Consequently, the relationship between ETC and geometric parameters can be derived as the following equation:

(2) $$\begin{array}{c} \left(\kappa_{yx}-\kappa_{yy}\cot\left(\theta_{2}\right)\right)\frac{\left|AO\right|}{\left|AD\right|}=0\\ \left(\kappa_{xx}-\kappa_{xy}\cot\left(\theta_{2}\right)\right)\frac{\left|AO\right|}{\left|AD\right|}=\kappa_{b}\\ \kappa_{o}=\kappa_{b}\phi /\xi \end{array}$$

where *κ_xx_*, *κ_xy_*, *κ_yx_*, and* κ_yy_* are the four entries of thermal conductivity tensors **κ**. *κ_b_* is the background conductivity. *ϕ* is a constant to be determined, *ξ* is a small positive number.

Upon selecting specific values that |*AO*| = 2|*AD*|, θ_1_ = −45°, and $\cot(\theta_{2})=-1/2$, we can simplify Equation (2) as follows:

(3) $$\begin{array}{c} 2\kappa_{yx}+\kappa_{yy}=0\\ 2\kappa_{xx}+\kappa_{xy}=\kappa_{b} \end{array}$$

We adopt the components of the temperature gradient from the literature^[^ ^48^ ^]^ and design with two materials (Copper, *κ*_copper_ = 400 Wm^−1^K^−1^, and PDMS, *κ*_PDMS_ = 0.16 Wm^−1^K^−1^). Calculating the thermal conductivity tensor of thermal metamaterial and background material, being $\kappa =[\begin{array}{cc} 13 & -13\\ -13 & 26 \end{array}]$ and $\kappa_{b}=[\begin{array}{cc} 13 & 0\\ 0 & 13 \end{array}]$, respectively. The theoretical simulated temperature distribution with the pure continuous materials manifests as depicted in Figure S1 (Supporting Information), thus achieving a thermal cloaking effect. Consequently, we can utilize the thermal conductivity tensor $\kappa =[\begin{array}{cc} 13 & -13\\ -13 & 26 \end{array}]$ as the targeted thermal conductivity tensor to complete follow‐up work, such as design the configuration of thermal cloaking metamaterial, set up datasets, and engage in DL models.

#### 2.1.2 Data‐Driven Design of TFCs with Configurable Mechanical‐Properties within Thermal Cloaking Carpet

The deep generative model has been widely employed in inverse design problems due to its capability to handle complex one‐to‐many mapping relationships between performance and structures. Here, we construct a deep generative model based on conditional variational autoencoders (CVAEs), capable of generating diverse structures, with its basic architecture following our previously proposed method.^[^ ^30^ ^]^ In our model, TFCs with their labels are compiled by the encoder into a series of learned distributions. During the microstructure generation process, the decoder randomly samples a series of latent variables within a given range under the guidance of these labels. As long as the sampled vectors conform to the learned distributions, they can become candidate solutions, thus achieving diversity in designing TFCs. Despite different configurations, many candidate solutions exhibit similar ETC compared to the target tensor with minimal error, controlled by the discriminative loss in the loss function. The model has been well trained on a diversified TFCs dataset, which is generated by topology optimization based on the BT‐independent design paradigm.^[^ ^49^ ^]^ (See Sections S1 and S3, Supporting Information, for more details).

Through the aforementioned model, we have crafted a diverse array of TFCs by targeting the thermal conductivity tensor $\kappa =[\begin{array}{cc} 13 & -13\\ -13 & 26 \end{array}]$ as our design objective (partially illustrated in Figure 1b). Nevertheless, the diversity within this finite dataset remains limited, necessitating further methods to expand the range of structural variations. This kind of expansion allows us to explore the mechanical properties and characteristics of these TFCs from a data‐driven perspective and leverage them effectively. To address this issue, we build a dynamic TFCs generator that primarily employs two data augmentation strategies. First, leveraging deep generative models to spawn new data by manipulating latent variables during generation. During this process, each sample in the dataset is encoded into the latent space. When generating new outputs, we can sample latent variables from the latent space and feed them into the decoder. Since each latent variable sample corresponds to a data label located in a specific region of the latent space, sampling from any point within that region theoretically allows for the generation of an infinite number of TFCs conforming to the target thermal conductivity tensor. Specifically, during TFCs generation, we can fine‐tune the values of one or more dimensions of a sampled latent variable, thereby obtaining entirely new output results. Second, we employ an iterative random shape perturbation algorithm^[^ ^50^ ^]^ for pixel‐level translations of 2D images. This algorithm performs translations on the input image based on provided shift images in the *x* and *y* directions. Subsequently, corresponding pixel coordinates in the input image are calculated based on the translated images. The pixel values corresponding to each pixel in the output image are computed via bilinear interpolation from the input image, and the data type of the output image is set to the same type as the input image. This algorithm transforms images through pixel‐level translations, finding applications in image processing and computer vision. Compared to the random material distribution method used in the literature,^[^ ^51^ ^]^ this perturbation approach offers higher chances of generating feasible TFCs with increased efficiency.^[^ ^52^ ^]^ With this algorithm, a series of new TFCs can be obtained by lightly perturbing the initial structures.

It is noteworthy to emphasize that our endeavor in augmenting the dataset via the aforementioned techniques is not solely to inflate its volume, but rather to enrich the diversity of the data. In contrast to prevalent data‐driven methodologies for metamaterial design, our approach expands the notion of “bigger data is better”^[^ ^53^ ^]^ in this field, by advocating that “while bigger data is better, the richness of data also matters”. By introducing latent variables encoding in metamaterial design, we provide interpretability to metamaterial generation, allowing us to focus primarily on the richness of data during design process rather than narrowly emphasizing data volume. Therefore, allocating more resources to explore diversified design freedoms expands the depth of the data space rather than its breadth. As shown in our work, demonstrates the ability to design multiple configurations of microstructures targeting specific thermal conductivity tensors at very low computational costs, enabling us to garner more choices and flexibility in designing thermal metamaterials.

Consequently, we can acquire a vast number of new samples, and build a database of diversified TFCs at almost zero computational cost. It should be noted, however, that while these new TFCs are obtained, we cannot guarantee the ideal ETCs for all samples. For defective results, they will be excluded in subsequent steps. As the newly acquired data lacks labeling, the distribution of thermal and mechanical properties within the database remains unclear. Annotating the continuously generated new data based on energy uniformization methods, i.e., predicting their ETC $\kappa =[\begin{array}{cc} \kappa_{xx} & \kappa_{xy}\\ \kappa_{yx} & \kappa_{yy} \end{array}]$ and equivalent stiffness tensors $C=[\begin{array}{ccc} C_{11} & C_{12} & C_{13}\\ C_{21} & C_{22} & C_{23}\\ C_{31} & C_{32} & C_{33} \end{array}]$, is computationally intensive. Therefore, drawing inspiration from the concept of self‐supervised learning strategy,^[^ ^43^ ^]^ we establish a convolutional neural network‐based property prediction model which can efficiently and accurately predicting the properties of these newly generated TFCs. Subsequently, the annotated samples are incorporated into the training dataset, and the model is retrained iteratively until its accuracy meets predetermined criteria. The annotated data thus serves as guidance for the subsequent selection of specific TFCs. (See Sections S2 and S3, Supporting Information, for more details).

Benefiting of the data augmentation, the variety of initial TFCs has been significantly expanded, leading to a diverse database annotated with properties. Through partial comparison between the original TFCs and the augmented database, as depicted in Figure S4 (Supporting Information), we observe a substantial enrichment of TFCs that are previously sparsely distributed in the property space, thereby broadening the available mechanical property spectrum. The subsequent challenge lies in uncovering the underlying patterns within such vast of data and flexibly accessing these structures. The most straightforward approach is to train a mechanical property prediction model to label all the microstructures. However, labeling the vast amount of continuously generated unlabeled data by using the prediction network, remains an extremely time‐consuming and computationally expensive task. Here, we employ two data dimensionality reduction algorithms to address this issue, Principal Component Analysis (PCA) and t‐distributed Stochastic Neighbor Embedding (t‐SNE). PCA is an unsupervised linear dimensionality reduction and data visualization technique employed for high‐dimensional data, aiming to reduce the dimensions of highly correlated data while preserving the global structure through principal components. However, PCA may lead to the crowding phenomenon during visualization. We combine PCA with the t‐SNE (an unsupervised nonlinear dimensionality reduction technique that aims to preserve the local structure of the data) to overcome this constraint. While the computational complexity of t‐SNE is relatively high, we mitigate this by first reducing the dimensionality using PCA and then applying t‐SNE for 2D or 3D visualization (Figure S5, Supporting Information), thus enhancing the presentation of data structure while reducing computational complexity. (See Section S4, Supporting Information, for more details).

**Figure 2:**
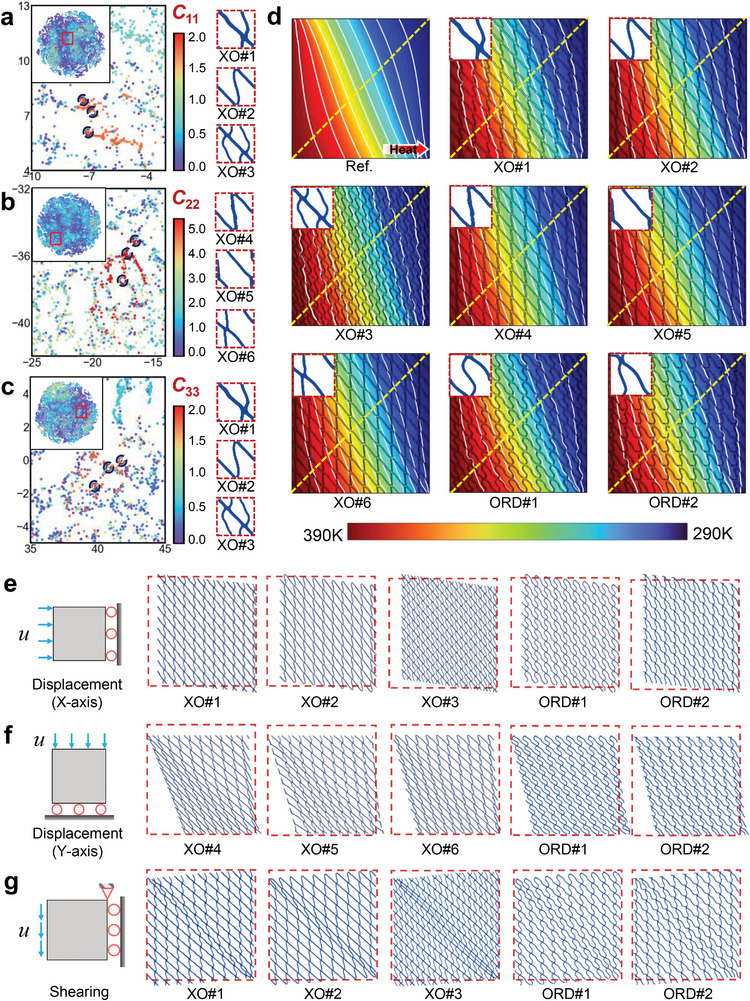
a–c) Feature extraction and latent space visualization. 2D distribution of data featured and colored by the magnitude of the stiffness tensors (*C*_11_, *C*_22_, and *C*_33_). Points are circled from the extracted latent distribution with *C*_11_≈2.0, *C*_22_≈5.1, and *C*_33_≈2.0, and the corresponding TFCs are displayed. The two axes correspond to the two dimensions after t‐SNE dimension reduction. The color legends denote the magnitude of each stiffness tensor. d) The temperature fields of theoretical and structural simulation of each TFC. The white lines are isotherms and the yellow dashed lines are observational lines. The color legend denotes the maximum and minimum temperature. e–g) Simulation analysis of designed TFCs under different boundary conditions (X‐displacement, Y‐displacement, and shearing).

Following dimensionality reduction and visualization, we generate distinct clustering cores by labeling different stiffness tensor components (*C*_11_, *C*_22_, and *C*_33_). As illustrated in **Figure** **2**a, color variations denote different tensor component magnitudes. TFCs with similar mechanical properties cluster together, enabling the selection of desired TFCs based on this 2D distribution. Since part of the generated data has already undergone mechanical property prediction and labeling, these data points are naturally distinguished in the latent space based on their property labels. This distinction helps outline the range and boundaries of the mechanical properties within the generated data. The mechanical property labels do not need to be precise numerical values; rather, they are used to locate clusters with specific extreme values in the latent space. This approach allows us to control the properties of the generated TFCs, which is why the mechanical properties of our designed TFCs are configurable. Therefore, for each set of components (*C*_11_ ≈ 2.0, *C*_22_ ≈ 5.1, and *C*_33_ ≈ 2.0), a series of TFCs can be considered as candidates, theoretically demonstrating superior mechanical performance compared to others. Subsequently, parts of these TFCs will be selected and undergo experimental simulations to validate the effectiveness of our method. Furthermore, it is noteworthy that our overarching strategy for generating configurable microstructures with mechanical properties is through data augmentation algorithms, which yield a vast amount of diversified data at minimal cost. Not all generated data need to meet our requirements (i.e., possessing target ETC and extreme mechanical properties), it suffices that such structures exist within the vast generated dataset and can be selected. While this method enables the generation of numerous TFCs with excellent mechanical properties, all resulting candidates must undergo selection via exclusion criteria: the structures must be intact and devoid of islands, and the thermal conductivity tensors of TFCs must align with the target tensors. As a result, the currently demonstrated dataset and its diversity are highly adaptable and feasible for identifying microstructures with extreme mechanical properties (such as elastic modulus, bulk modulus, and shear modulus) or specific Poisson ratios. It is also capable of designing microstructures with specific elastic moduli within a certain range. However, it must be acknowledged that designing microstructures with specific properties across a broader property space and boundaries would require further expansion of data diversity.

#### 2.1.3 Simulated Verifications of TFCs with Configurable Mechanical‐Properties

Among the above‐generated extraordinary (XO) TFCs, the top three are selected for each group of *C*_11_, *C*_22_, and *C*_33_, for a total of nine TFCs. It is worth noting that there exist repeated units in groups *C*_11_ and *C*_33_, which exhibit exceptionally large values of *C*_11_ and *C*_33_. Therefore, these TFCs are merged and denoted as XO#1, XO#2, and XO#3 for subsequent mechanical simulations involving X‐displacement and shearing tests. The three TFCs from group *C*_22_ are denoted as XO#4, XO#5, and XO#6 for mechanical simulations in the Y‐displacement. To demonstrate the effectiveness of our method, we make a 10 × 10 periodic structure of these TFCs and set the size of periodic structure as 60 mm × 60 mm. Then, we evaluate their thermal and mechanical performance by finite‐element method (FEM) simulations in commercial software COMSOL Multiphysics 6.1. Additionally, we randomly select two ordinary (ORD) TFCs (designated as ORD#1 and ORD#2) for comparison with the extraordinary TFCs.

**Figure 3:**
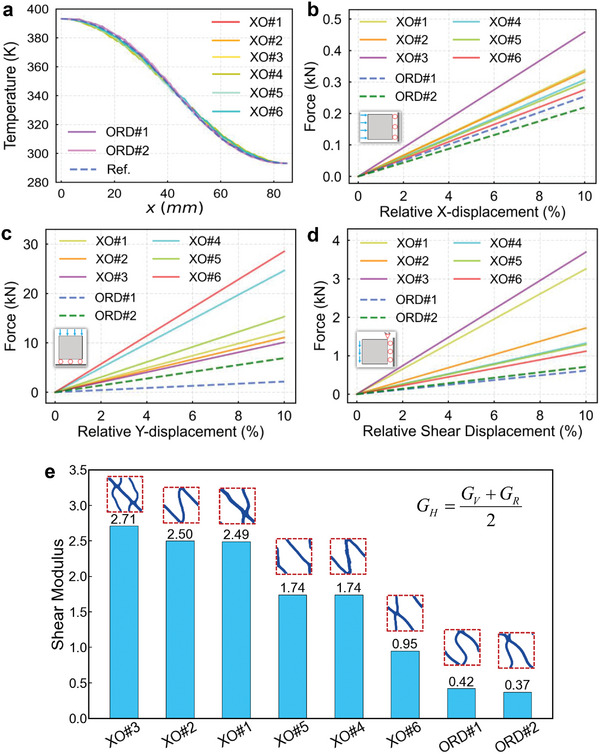
a) Simulated results of measured temperature along the observation lines. The dashed blue line represents the reference. b–d) The simulated force‐displacement graphs under different boundary conditions. All solid lines represent extraordinary TFCs, and dashed lines represent ordinary TFCs. The two axes are relative displacement percentage and reaction force. e) Bar graph of the shear modulus for all TFCs.

For thermal simulations, we apply constant temperatures on the boundary and set adiabatic boundaries parallel to the temperature gradient. The thermal conductivities of two materials are set as 400 and 0.16 Wm^−1^K^−1^, respectively. As can be seen from Figure 2d, the temperature gradient of each TFC is consistent with the reference temperature gradient in the continuous medium (the thermal conductivity tensor is set to be the same as the target thermal conductivity tensor). Additionally, we plot temperature profiles along yellow observational lines in each simulated temperature field to compare them with the reference temperature, as depicted in **Figure** **3**a. The simulation results illustrate that the macroscopic equivalent thermal conductivity tensor of designed TFCs can be considered to meet the target thermal conductivity tensor.

Moreover, we can conduct additional simulations to validate the mechanical performance of these TFCs. Figure 2e–g illustrates the application of three different boundary conditions utilized in our simulations. In each simulation scenario, we apply a maximum displacement of *u*_max_ = 6 mm at the boundaries of the periodic structure to observe the magnitude of the force that they can withstand under the same deformation. By employing boundary probes, we obtain force‐displacement graphs for each structure, as shown in Figure 3b–d. We observe that the stiffness (slope of the force‐displacement curve) of all designed extraordinary TFCs surpasses that of the ordinary structures. This indicates that under equivalent boundary conditions, our designed TFCs can withstand larger loads, exhibiting significantly stronger structural mechanical performance compared to the undesignated TFCs.

Furthermore, due to the anisotropic nature of the TFCs we design, it is not straightforward to represent the shear modulus of the structures using a single parameter. In such cases, it is typically necessary to account for the anisotropic properties and may require the use of more complex methods to compute the shear modulus. Here, we employ the Voigt‐Reuss‐Hill (VRH) method^[^ ^54^ ^]^ to approximately evaluate the elastic property of designed anisotropic TFCs. The VRH method combines the Voigt method and the Reuss method, and often involves the Hill averaging scheme. The calculation formulas are as follows:

(4) $$\begin{array}{c} G_{V}=\frac{1}{8}\left(C_{11}+C_{22}-2C_{12}+4C_{33}\right)\\ G_{R}=2{\left(\frac{1}{C_{11}}+\frac{1}{C_{22}}-\frac{2}{C_{12}}+\frac{1}{C_{33}}\right)}^{-1}\\ G_{H}=\frac{G_{V}+G_{R}}{2} \end{array}$$

where *C_i_*_,_ *_j_* are the stiffness constants in the respective directions. *G_H_* is the estimated shear modulus using the VRH method.

We calculate the estimated shear modulus of all TFCs by applying Equation (4) and summarize them in a bar graph, as depicted in Figure 3e. The TFCs designed with extraordinary mechanical properties exhibited significantly higher shear modulus compared to the ordinary ones, averaging more than five times higher. These results validate the effectiveness of the proposed method, demonstrating its capability to efficiently design TFCs with outstanding mechanical properties and specific ETCs.

### 2.2 Simulated and Experimental Demonstrations for Designed Thermal Cloaking Carpet

**Figure 4:**
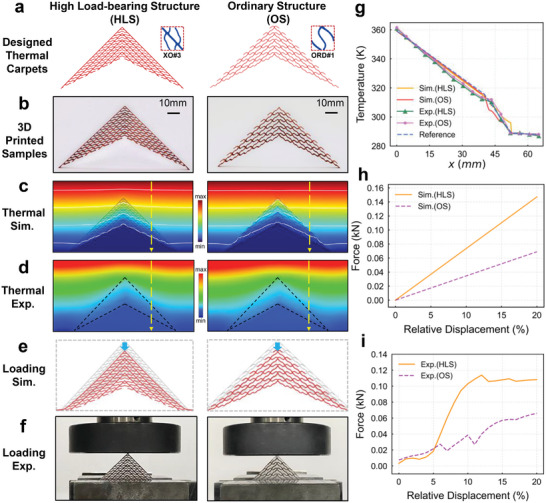
Simulated and experimental results of designed thermal cloaking carpets. a) Designed thermal carpet configuration for two structures. b) 3D‐printed thermal carpets (without the PVC cover and PDMS fillings). c,d) Simulated and experimentally measured temperature fields of two thermal carpets under heat flow from top to bottom. White lines are isothermal lines and the yellow dashed lines represent the observation lines. e,f) Simulated and experimental loading test. g) Measured temperature values along the observation lines. The dashed blue line represents the reference. h,j) The force‐displacement graphs for simulation and experiment. The solid lines represent the high load‐bearing structure, and the dashed lines represent the ordinary structure. The two axes are relative displacement percentage and reaction force.

After the comprehensive elucidation of design process and simulation verification outlined above, we proceed to assemble the designed TFCs into design region to form a complete thermal metamaterial. Through simulation and experimentation, we validate the functionality of designed thermal cloaking carpets. Specifically, we select one of each from the extraordinary and ordinary TFCs (XO#3 and ORD#1) to assemble them into thermal cloaking carpets with high load‐bearing structure (HLS) and ordinary structure (OS), which are subsequently prepared using 3D printing technology into copper structures of 5 mm thickness, as depicted in **Figure** **4**a,b. Subsequently, the liquid PDMS is filled into the voids of the printed structures, followed by PDMS deaerating and curing, obtaining the formation of complete thermal cloaking carpets. Figure 4c,d presents the simulation and experimental results of these two thermal cloaking carpets. It can be observed that both carpets exhibit excellent thermal cloaking functionality under the top‐down heat flow, effectively counteracting the interference of the object with the background temperature field and forming horizontal isotherms, which has demonstrated a significant difference in temperature distribution when the thermal cloak carpet is absent (see Figure S7, Supporting Information), highlighting the thermal carpet's effectiveness in achieving thermal cloaking. Furthermore, by setting temperature observation lines for comparison, as shown in Figure 4g, the temperature gradient along this line closely matches the ideal scenario, further indicating the excellent thermal cloaking characteristics of designed thermal carpets.

Besides, we conduct mechanical simulation and experimentation on the designed structures, as depicted in Figure 4e,f. Placing the thermal carpets on a compression platform, we subject them to a maximum relative displacement of 20% in a single direction. The force‐displacement graphs from simulation and experimentation are respectively shown in Figure 4h,i. It is evident that the load‐bearing capacity of the robust structures far exceeds that of conventionally designed structures. However, since non‐linear effects, friction, stress concentration, out‐of‐plane bending, and additive manufacturing integrity have not been considered, discrepancies between simulation and experimental results may arise. For the experimental result of HLS, when the displacement is under 5%, point contact in the loading and constraint conditions leads to significant relative deformation, not reflecting true mechanical performance. Between 5% and 12% displacement, the structure transitions to surface contact, achieving a stable state and demonstrating high stiffness with a true linear mechanical response. Beyond 12% displacement, significant deformation and geometric nonlinearity cause a nonlinear response. Thus, displacements under 5% and over 12% indicate instability, while the stable state between 5% and 12% aligns with simulation results, accurately reflecting the true mechanical properties. In contrast, the OS structure exhibits lower overall stiffness and softer characteristics. Under initial loading, the contact points (some of which are solid PDMS) deform, the structure reaches a stable state rapidly. Its softer structural properties allow it to maintain an approximately linear mechanical response within a 20% relative displacement range, showing a force‐displacement relationship that closely matches the simulation results. Therefore, combining all experimental and simulation results, we can conclude through qualitative analysis that the HLS possesses superior structural strength and load‐bearing capacity, while maintaining excellent thermal performance even under load‐induced deformation. This demonstrates that our design methodology effectively enables the design of thermal metadevices with specific thermal functionalities, while their mechanical properties can be deliberately configured. (see Sections S5 and S6, Supporting Information, for more details).

## 3 Conclusion

In summary, we propose a data‐driven approach leveraging AI techniques for the design and fabrication of thermal metamaterials with configurable mechanical properties, emphasizing thermal cloaking functionality and load‐bearing capability. Our methodology harnesses the power of deep generative models, combined with innovative data augmentation strategies and dimensionality reduction visualization techniques, to cluster microstructures with analogous geometries together. Sampling specific clusters in the latent space enables the regeneration of new designs from latent variables, thus uncovering numerous designs with distinct geometric configurations yet satisfying the same thermal property requirements, addressing the inherent complexities of metamaterial design. By enhancing the diversity of the acquired dataset and uncovering latent patterns within vast data spaces, we have further broadened the diversity selection and significantly enhanced design freedom.

Consequently, one can successfully engineer thermal metamaterials capable of seamlessly integrating thermal functionalities while offering tailored mechanical properties. Through extensive simulations and experimental validation, we demonstrate the effectiveness of our approach in crafting TFCs with exceptional mechanical performance and specific ETC. Our study not only contributes to advancements in the design of thermal metamaterials but also holds promise for various applications. The multifunctionality and adaptability of our approach pave the way for further exploration of additional thermal functionalities and physical domains, fostering innovation and breakthroughs in the field of metamaterial research. Overall, our study underscores the transformative potential of data‐driven approaches and artificial intelligence techniques in revolutionizing the paradigm of metamaterial design, offering new insights and pathways for developing complex metamaterials with extraordinary capabilities.

## 4 Experimental Section

The thermal cloaking carpets were printed with the size of ≈100 mm × 50 mm × 5 mm and were fabricated using pure copper powder laser sintering. To validate the thermal performance of the carpets consistent with numerical simulations, a 130 mm × 70 mm × 5 mm high thermal conductivity silicone plate (13 W m^−1^ K^−1^) was employed as the background material. In order to generate a uniform temperature gradient, heating and cooling modules were installed at both ends of the background plate, and the entire outer surface was covered with 0.1 mm thick polyvinyl chloride (PVC) tape to ensure uniform infrared emissivity. The temperatures were set to *T*_max_ = 360 K and *T*_min_ = 287 K to match the numerical simulations with experiments. After reaching a steady state, a thermal infrared camera (FOTRIC246 M) placed above the metadevice captured the temperature field as thermal infrared images, which were analyzed using thermal imaging analysis software, AnalyzIR. The thermal experimental setup is illustrated in Figure S6 (Supporting Information). For the mechanical performance validation of the thermal cloaking carpets, the carpets were placed within an unfixed fixture, allowing free movement, and subjected to testing on an autograph universal testing machine (AG‐IC 100 kN). The mechanical experimental setup is depicted in Figure S8 (Supporting Information).

## Conflict of Interest

The authors declare no conflict of interest.

## Supporting information

Supporting Information

## Data Availability

The data that support the findings of this study are available from the corresponding author upon reasonable request.
