# Supplementary material for: Thermal Metamaterials with Configurable Mechanical Properties
Source: Adv Sci (Weinh). 2024 Sep 3;11(40):2406116. doi: 10.1002/advs.202406116 (PMC11516070; doi:10.1002/advs.202406116)
Supplement: Supplementary file 1 — Supporting Information [file ADVS-11-2406116-s001.docx]

Supporting Information

**Thermal Metamaterials with Configurable Mechanical Properties**

Yihui Wang, Wei Sha, Mi Xiao,* and Liang Gao*

1. **Diversified TFCs Dataset**

The training set of the model originates from topology optimization computations based on the BT-independent design paradigm. Utilizing this methodology, we aim to attain the desired thermal conductivity tensor by considering two constituent materials: Copper (Pure Copper, thermal conductivity *κ*_copper_ = 400 Wm^−1^K^−1^) and polydimethylsiloxane (PDMS, thermal conductivity *κ*_PDMS_ = 0.16 Wm^−1^K^−1^). This enables the determination of configurations for TFCs (as a 2D image of resolution 60 × 60). Given the sensitivity of topology optimization to initialization parameters, subtle adjustments in the initial conditions afford a diverse array of configurations. Leveraging this characteristic, we systematically vary the size, quantity, and spatial distribution of initial voids to generate a comprehensive spectrum of TFC configurations. Under omnidirectional heat flux, these TFCs maintain the ideal macroscopic ETCs. Hence, we compile a dataset comprising 189 sets of original TFCs and compute their respective ETCs and equivalent stiffness tensors utilizing an energy-based homogenization technique. Despite the computational intensity involved in data acquisition and labeling, the resultant dataset remains limited, precluding robust model training. Employing data augmentation strategies offers a viable approach to expand the dataset, thus bolstering model performance. By incorporating intermediate results derived during the topology optimization iterations, we amass a dataset comprising approximately 20,000 initial samples for initial dataset. This strategy effectively mitigates concerns related to overfitting. Furthermore, certain TFCs are excluded from the dataset due to structural inadequacies such as fracturing, isolation, or the presence of checkerboard patterns.

1. **Forward Prediction Model Based on Convolutional Neural Network (CNN)**

As depicted in Figure S2a, our proposed convolutional neural network (CNN)-based forward prediction model encompasses multiple hidden layers, systematically divided into three categories: convolutional layers (Conv), pooling layers (Pooling), and fully connected layers (FC). Convolutional layers undertake the task of identifying distinct patterns within TFCs by systematically traversing the entire input structure with a set of filters, each composed of 5×5 kernel size, and computing the dot product between the filter weights and input pixel values. Throughout the training, these filter weights are optimized to refine feature extraction. Consequently, the CNN delineates and extracts localized features from various image regions, gradually transitioning from detecting low-level features such as edges in the initial Conv layer to discerning higher-level features in subsequent layers. This hierarchical feature extraction process culminates in a holistic understanding of the input dataset. Additionally, pooling layers, adjunct to each convolutional layer, contribute to dimensionality reduction of feature maps, effectively mitigating the risk of overfitting. Lastly, the model incorporates fully connected layers, wherein each neuron is intricately linked to all labels inferred from the preceding convolutional layers. By constructing this forward prediction model, we facilitate the generation of precise real-valued predictions, thereby encapsulating the thermal conductivity tensors of input samples with enhanced accuracy and reliability. Partial instances of property prediction are illustrated in Figure S3b, where 2D images of TFCs serve as inputs, yielding predictions for specific entries of thermal conductivity tensors. The outcomes demonstrate the model's capability to efficiently and accurately forecast property values within an acceptable margin of error. Additionally, we develop a mechanical properties predictor with the same network architecture and trained it using a transfer learning strategy. This model is used to label the mechanical properties of the continuously generated unlabelled data. It is important to note that it is not necessary to label all the microstructures, as this is a time-consuming task. We only need to randomly select 25% of the microstructures for mechanical property labeling. Then, using dimensionality reduction algorithms for clustering that can identify clusters with extreme value labels.

1. **Deep** **Generative Model with Self-supervised Strategy**

As shown in Figure S3, we develop a deep generative model based on Conditional Variational Autoencoder (CVAE). This model efficiently transforms complex geometric structures into continuous and organized latent space representations. During training, we leveraged a precomputed database of TFCs, aiding the model in better learning the data representation. The encoder consists of a series of layers, including 2D convolutional layers, batch normalization layers, and fully connected layers, capable of converting inputs into a simplified 20-dimensional latent representation. The decoder section possesses a structure opposite to the encoder, mapping the latent representation back to the original data space. A variety of TFCs can be obtained by continuous sampling in characteristic areas. Furthermore, to save the burden of data collection and annotation, we introduce a self-supervised learning strategy, where unlabeled data is generated online and fed into the model together with pre-collected labeled data during the training process. For unlabeled data, the decoder takes the predicted property from the prediction model as pseudo-labels. Thus, total loss function to train the deep learning model is given by:

(S1)

Where the Kullback-Leibler (KL) divergence loss assesses the difference between the distribution produced by the encoder and the assumed prior distribution of *z*. The reconstruction loss determines the fidelity with which the decoder reconstructs the input structure from its latent variable and associated thermal conductivity tensor. A discrimination loss is incorporated to compare the predicted attribute of the generated TFC with the desired tensor, using mean squared error (MSE) as the metric.

In configuring the model training parameters, we initiate random shuffling and partition our database into a training set comprising 16,000 TFCs and a separate test set containing 4,000 TFCs. Employing a batch size of 100, the model undergoes training for 200 epochs until convergence or 10 epochs without any discernible improvement, upon which the training process terminates. Our model training occurs on a workstation equipped with an NVIDIA GeForce RTX 3080, boasting 10 GB of memory capacity.

1. **Dimensionality Reduction and Data Visualization**

PCA is a widely employed unsupervised technique for linear dimensionality reduction and data visualization, particularly suited for high-dimensional datasets. PCA operates on the premise of transforming the original dataset into a new set of orthogonal vectors known as principal components, with the objective of minimizing the dimensionality while preserving the overall structure of the data. However, PCA is inherently limited in its ability to capture the local structure of the data, which may result in the loss of important information, a phenomenon commonly referred to as the "crowding problem". In addition to its utility in conserving memory, one of the primary motivations for dimensionality reduction techniques is to facilitate data visualization. By projecting high-dimensional data onto a lower-dimensional space, typically 2 or 3 dimensions, we can visually discern the underlying patterns and relationships within the database. Despite its effectiveness, PCA may not always provide satisfactory visual representations due to the crowding problem. To address this issue, an alternative approach involving t-Distributed Stochastic Neighbor Embedding (t-SNE) is introduced. Unlike PCA, t-SNE is a nonlinear dimensionality reduction technique that focuses on preserving the local structure of the data. By minimizing the Kullback-Leibler (KL) divergence between the high-dimensional and low-dimensional distributions of data points, t-SNE aims to faithfully represent the neighborhood relationships in the lower-dimensional space. While t-SNE offers superior preservation of local structure compared to PCA, it comes with increased computational complexity, especially when dealing with large-scale datasets or high-dimensional data. To mitigate this computational burden, a pragmatic approach is adopted: first, reduce the dimensionality of the data to approximately 10 dimensions using PCA. Subsequently, apply t-SNE to further reduce the dimensionality to 2 or 3 dimensions for visualization purposes. This integrated methodology not only addresses the computational challenges associated with t-SNE but also ensures the retention of critical data features, thereby enhancing the interpretability of the data structure in the lower-dimensional space.

1. **Thermal Performance Stability of Thermal Carpet after Deformation**

For linear elastic problems, to maintain consistency with the mechanical simulations and experiments in the manuscript, we assume a maximum deformation of the structure as 20% relative to its original height (50 mm). We have conducted load tests on the structure with relative displacement of 10%, 16%, and 20%, respectively. Subsequently, thermal simulations have been performed on the deformed structure under the same thermal boundary conditions consistent with the undeformed case (Figure S9a). The deformed structure and thermal simulation are illustrated in Figures S9b-d. By comparing the simulation results, it is observed that the white isothermal lines in the background material remain horizontal, indicating that the thermal carpet eliminates the influence of object on the background temperature field. Additionally, temperature observation lines (yellow dashed lines) have been placed in the same positions, with their temperature profiles shown in Figure S9e. It can be seen that in the background region, the temperature curves of the deformed structures are similar to the reference temperature, while in the cloaked region, the temperature remains constant.

Overall, the simulation results demonstrate that the deformed structures continue to exhibit excellent thermal cloaking performance. This validates the thermal performance stability of our designed thermal cloaking carpet under load and deformation conditions.

1. **Comparison of loading tests for different thermal carpets.**

The assembling process of thermal carpets, as shown in Figure S10. The microstructures are fitted into the design domain after array and mirror replication. From this assembly process, it can be observed that some microstructures, such as XO#1 and XO#3, have material configurations along the diagonal, resulting in boundaries parallel to the design region upon sectioning, rather than artificially constructed boundaries. Additionally, we find that all HLS structures exhibit parallel structural features internally, which is a direct manifestation of the excellent structural characteristics of our designed metamaterials on a macroscopic scale. Certainly, in practical engineering applications, designers may modify the structural shape (as shown in Figure S11a, where Copper is added at the boundaries of the thermal cloak) to enhance structural stiffness. However, it is important to note that such modifications fall under post-processing and diverge significantly from the original theoretical design intent of our study. Moreover, we cannot guarantee that the modified structures will retain their thermal cloaking performance.

To further investigate which structure has the strongest load-bearing performance, we conduct the same mechanical simulations on HLS-XO#1, HLS-XO#2, and OS-Rep. The results, shown in Figure S11b-e, indicate that HLS-XO#3 indeed has the best load-bearing performance, validating our previous conclusion that XO#3 is the optimal choice. Comparing the results of HLS-XO#1, HLS-XO#2, OS-Rep., and OS-Orig., also reveal that the presence of material at the boundaries does not significantly impact the compressive strength of the structure. The structural strength is primarily determined by the configuration characteristics of the structure, underscoring the necessity of stiffness design in our structures.

**
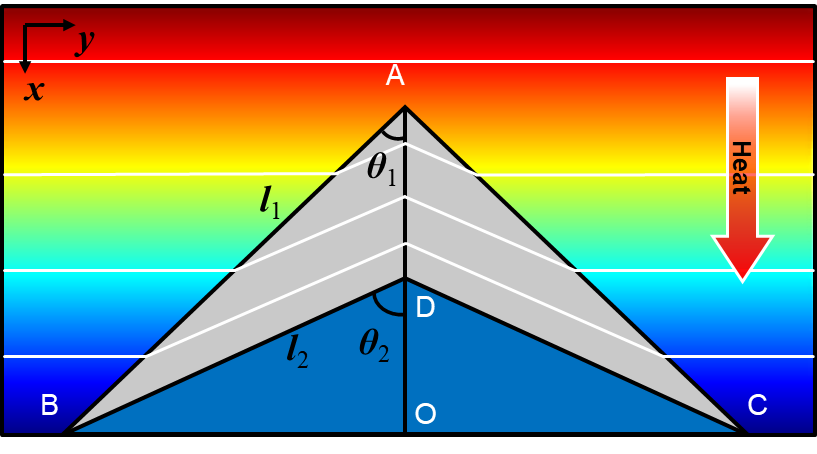
**

**Figure S1**. Design of thermal cloaking carpet based on regionalized scattering cancellation method. The design area is divided into different regions through the interface *l*_1_ and *l*_2_. The rainbow gradient area represents the background with heat flow from top-down, the blue area represents the thermal functional area, and the gray area represents the thermal metamaterial. The white lines are isotherms.

**
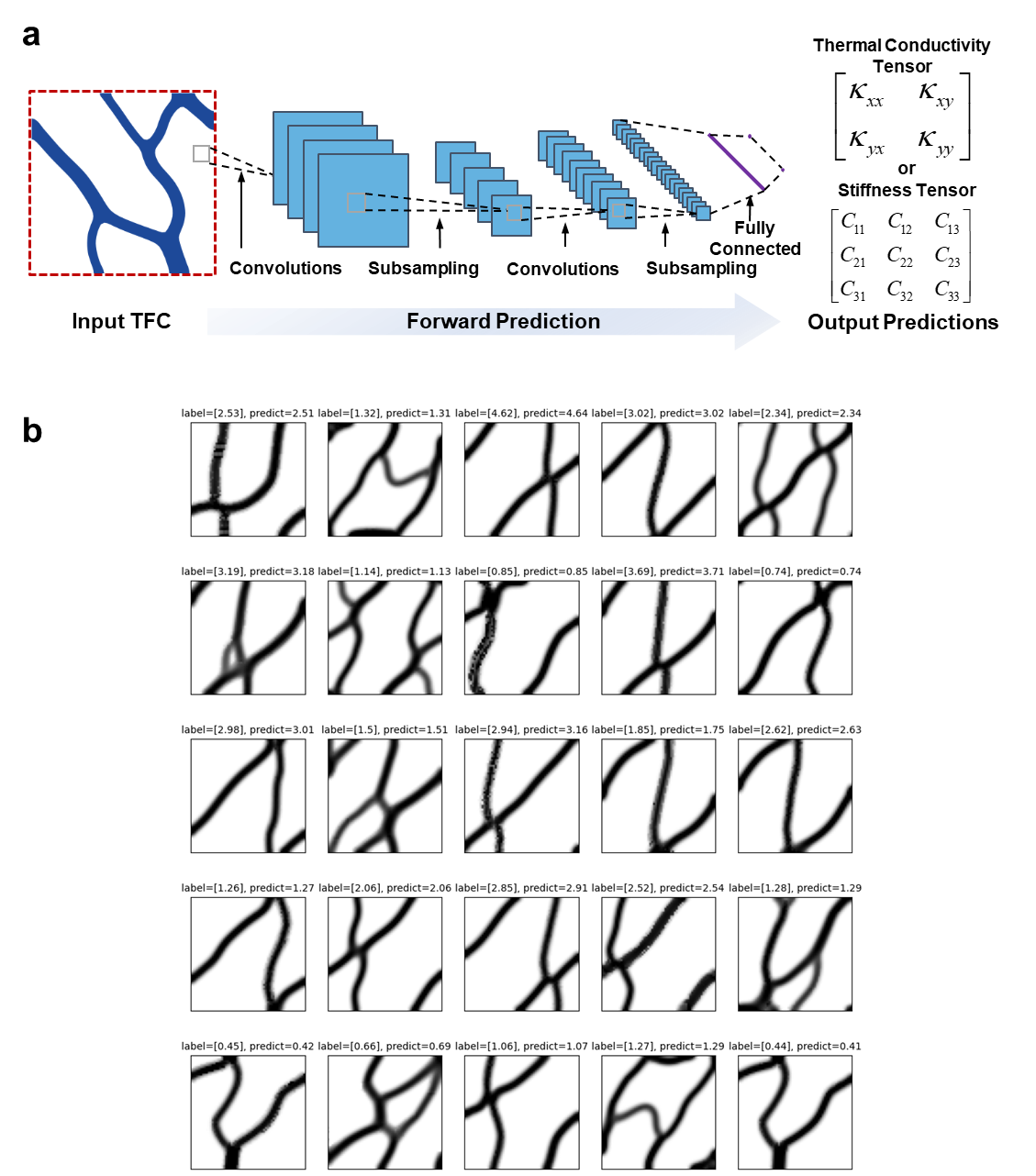
**

**Figure S2**. a) CNN-based forward prediction model comprises convolutional, pooling, and fully connected layers. Convolutional layers identify patterns, pooling layers reduce dimensionality, and fully connected layers integrate information. b) examples of prediction.

**
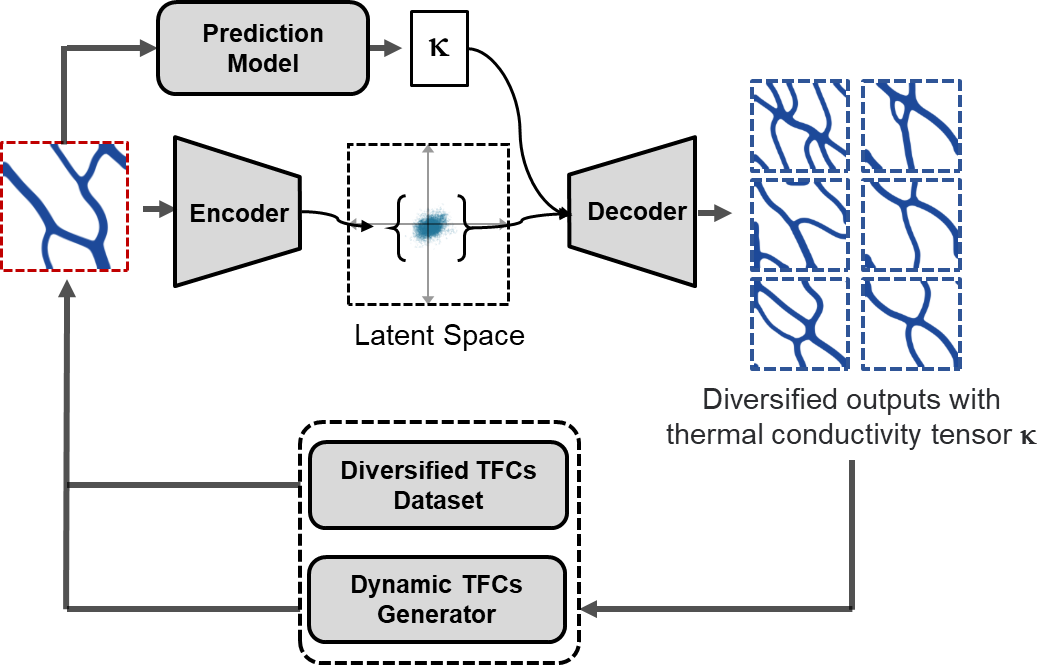
**

**Figure S3**. The schematic diagram of the deep generative model. Comprising an encoder and a decoder, the encoder encodes TFCs from the dataset into a latent space, while the decoder samples within specific regions corresponding to thermal conductivity tensor, thereby generating diverse structures of TFCs.


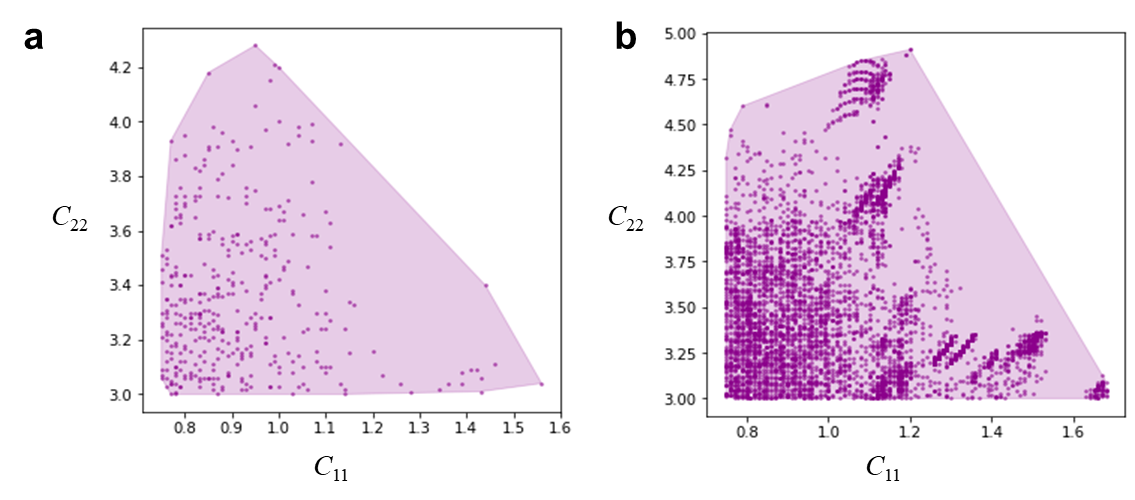


**Figure S4**. Comparison of property space before and after data augmentation. The two axes correspond to *C*_11_ and *C*_22_.

**
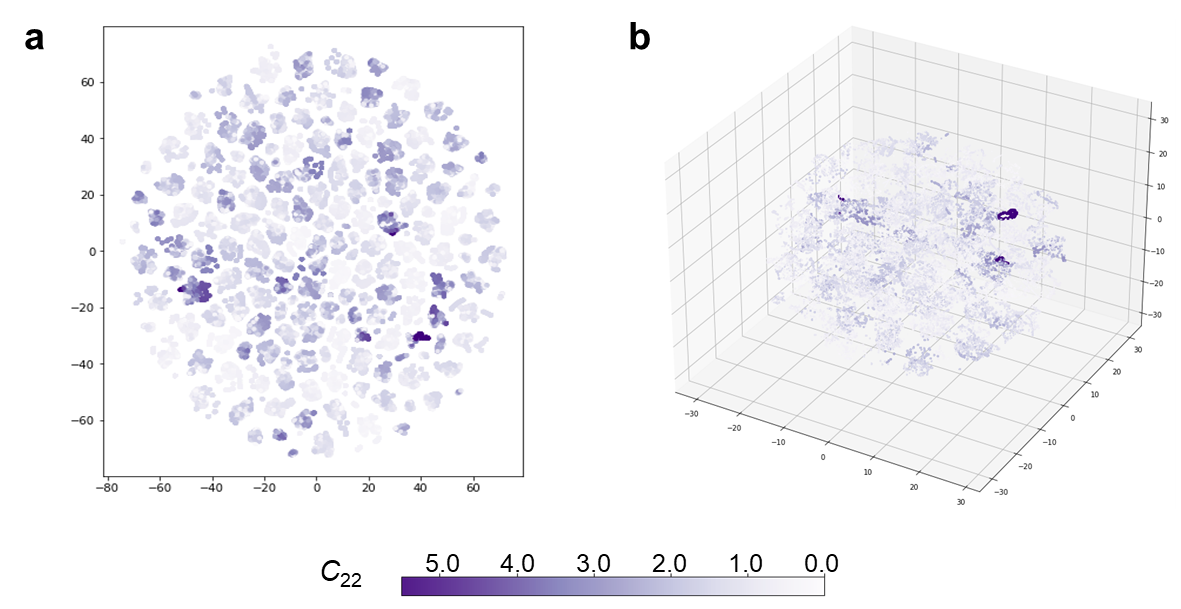
**

**Figure S5**. Visualization graphs in 2D and 3D space achieved through t-SNE dimensionality reduction. The axes correspond to the two dimensions and three dimensions after t-SNE dimension reduction. The color legends denote the magnitude of stiffness tensor *C*_22_.

**
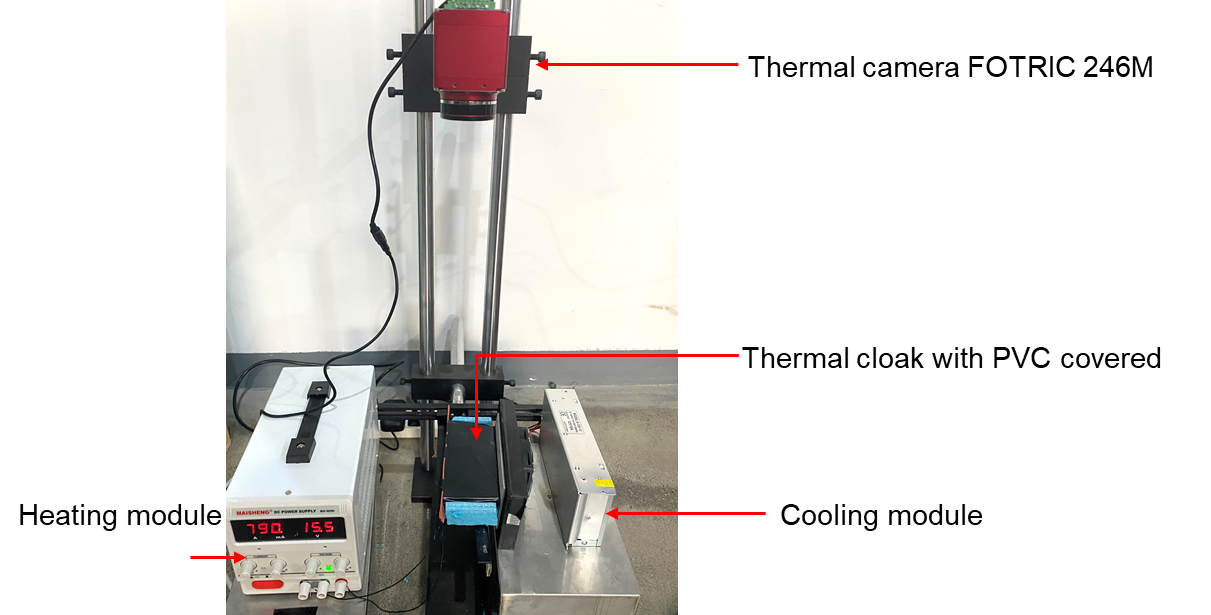
**

**Figure S6**. Experimental setup consisting of the thermal carpet, heating&cooling module and thermal camera. To ensure the same infrared emissivity, the surface of thermal cloak was covered by 0.1mm thick PVC black tape.

**
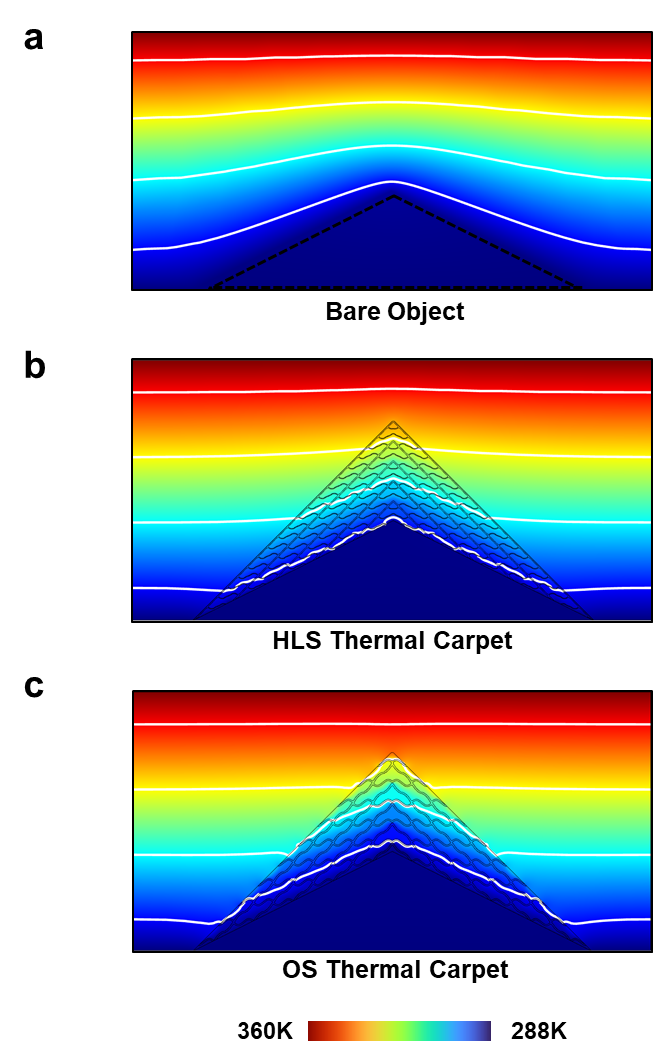
**

**Figure S7**. The thermal simulation comparison of bare object, HLS thermal carpet and OS thermal carpet.

**
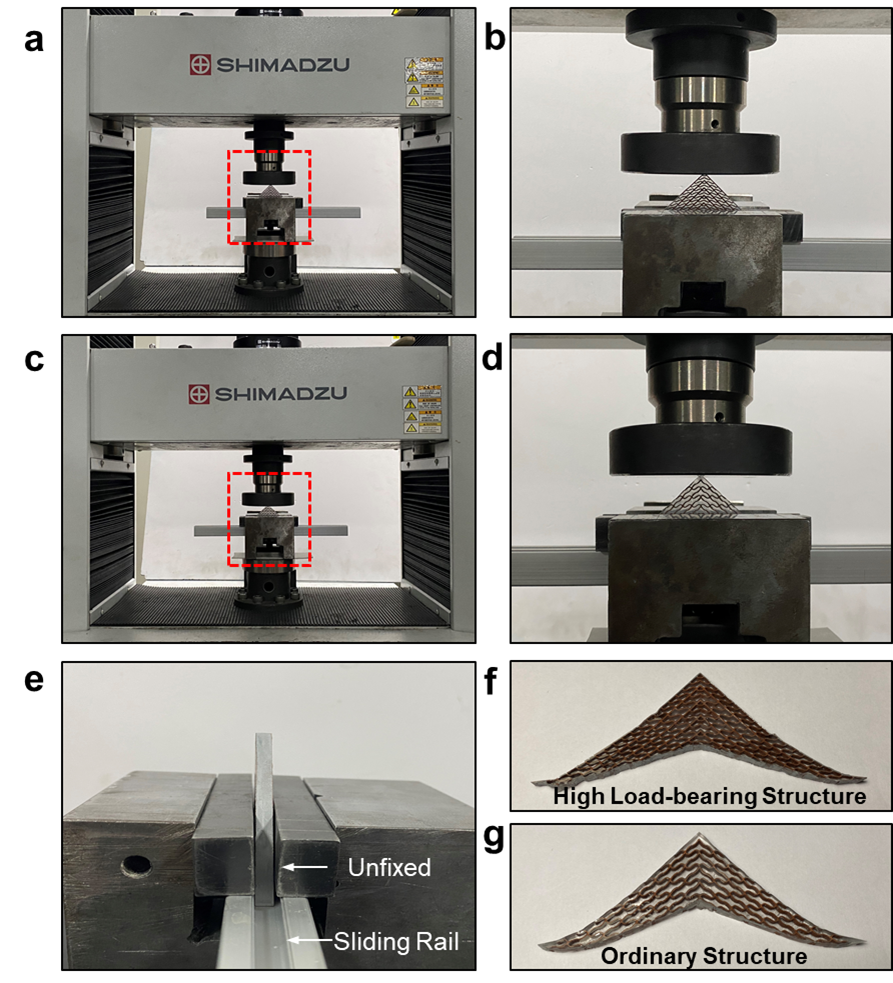
**

**Figure S8**. a-d) The scene of compression experiment. e) The object is not fixed by the fixture, but is loose and can move freely between the fixture along the sliding rail. f,g) The deformed thermal carpets after experiment.


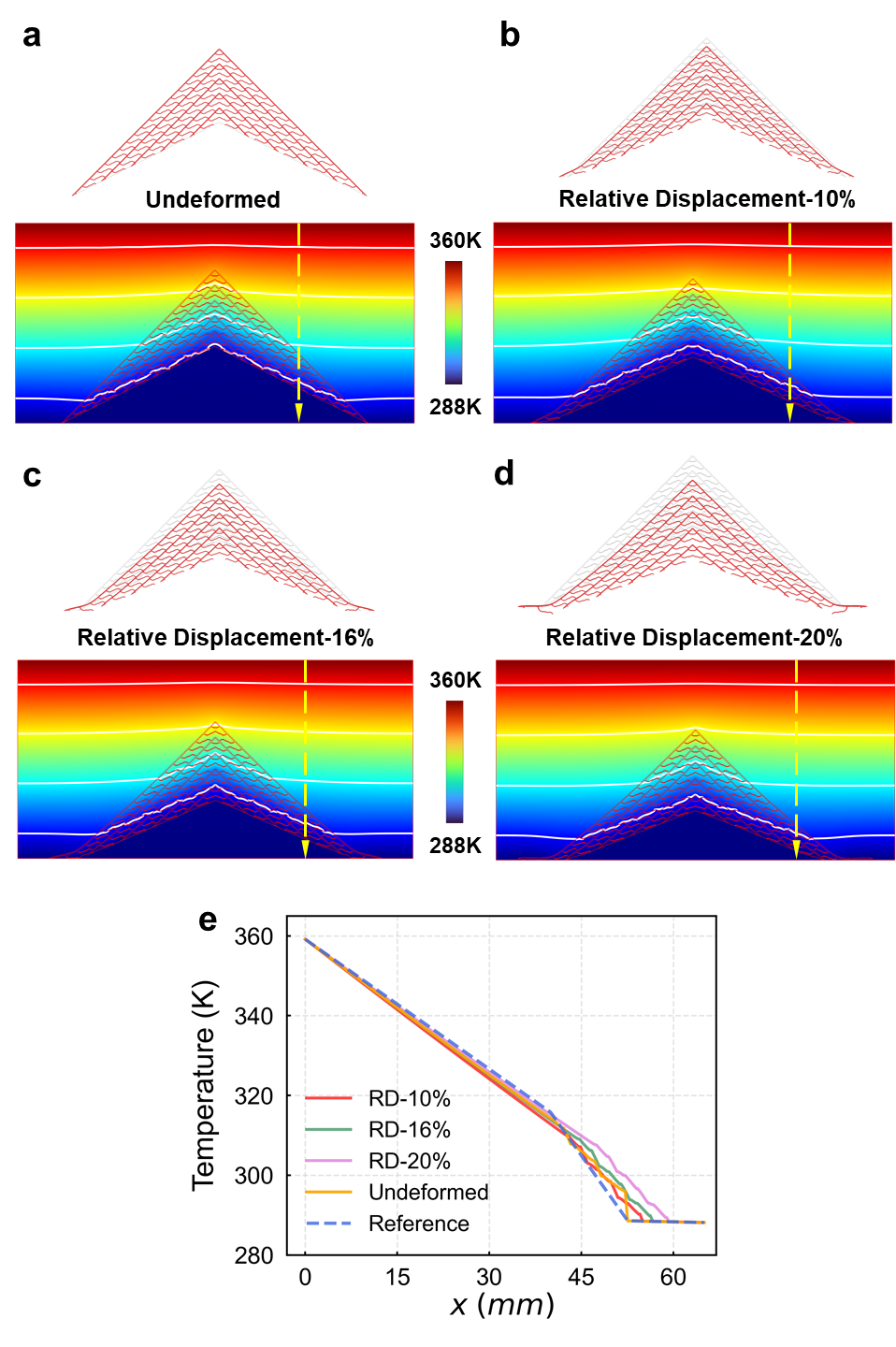


**Figure S9**. a-d) The deformed structures and thermal simulation results. e) Measured temperature values along the observation lines. The dashed blue line represents the reference.


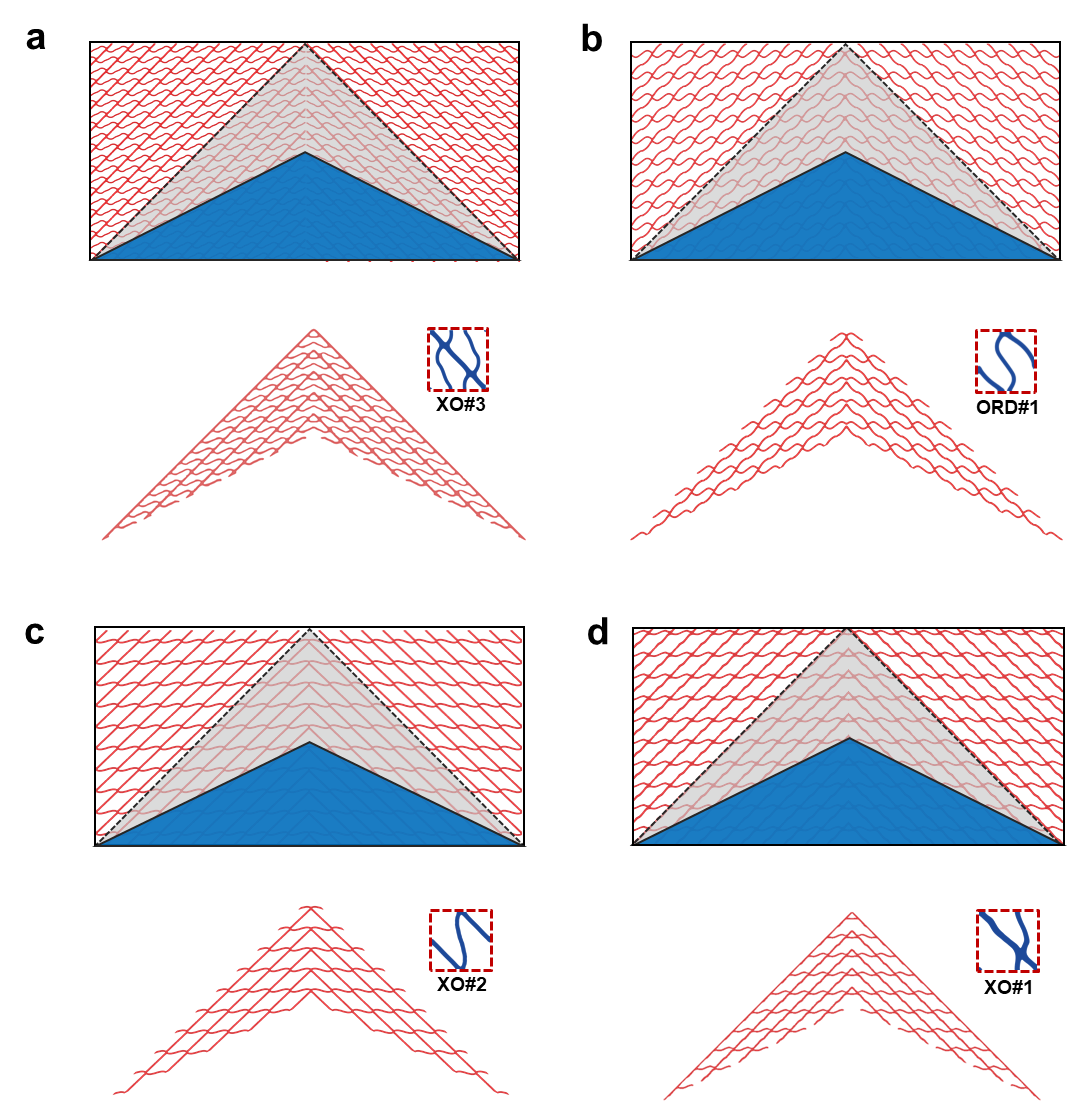


**Figure S10**. The assembling process of thermal carpets.


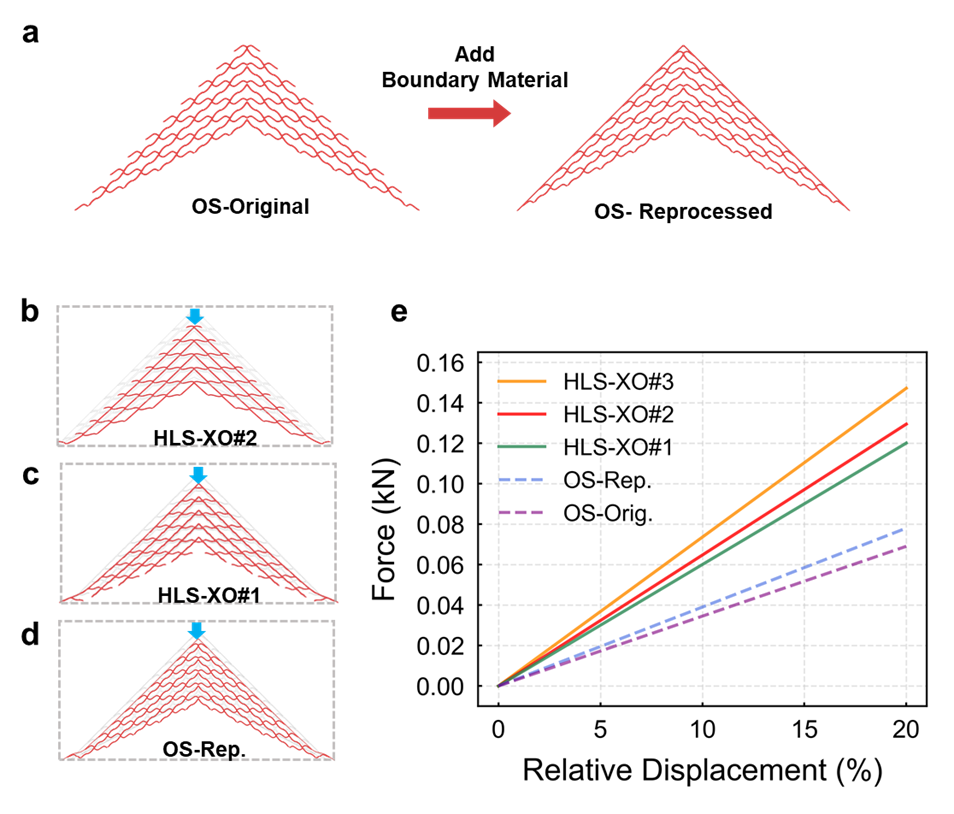


**Figure S11**. a) The reprocessed thermal carpet (OS). b-d) Loading test simulations for additional thermal carpets. e) The force-displacement graphs for simulations of all thermal carpets.
